# Characteristics of Animal-assisted Interventions in the state of Lower Saxony, Germany, with a focus on hygiene in health care facilities

**DOI:** 10.1016/j.onehlt.2023.100620

**Published:** 2023-08-22

**Authors:** Sonja Wolken, Johannes Dreesman, Dagmar Rocker, Cornelia Henke-Gendo

**Affiliations:** Public Health Agency of Lower Saxony, Hannover, Germany

**Keywords:** Animal-assisted intervention, Animal-assisted therapy, Therapy dog, Assistance dog, Hospital hygiene, Animal welfare

## Abstract

Animal-assisted Interventions (AAIs) are becoming increasingly popular. To date, information on the extent of AAIs in Germany is limited. With a focus on infection control measures in health care facilities (HCFs), two studies were conducted in Lower Saxony to gain knowledge about the structure, characteristics and frequency of AAIs. An online survey among AAI providers identified dogs as the most important animal species in AAI, which mainly operated in educational facilities (53%) and/or on own property (46%). Twenty-nine percent offered their services in HCFs. The majority (55%) of the animal handlers was highly trained in AAI, but their awareness of hygiene and infection control measures to prevent zoonotic disease transmission was limited. Nineteen percent of animal handlers dewormed dogs only when faecal examinations were positive and 13% of dogs received ectoparasiticides only when infestations were present. Raw meat diets were frequent (82%). There was little awareness among animal handlers about the possibility of a zoonotic transmission from the client to the animal. Thus, handling of therapy dogs often reflected that of a “normal” pet ownership and did not always account for the special situation in HCFs. A telephone survey in 148 hospitals showed that 28% of the hospitals had experiences with animal-assisted therapies or animal visits, but 22% of these were lacking regulations on handling these animal contacts. While 28% of all hospitals had regulations for assistance dogs only 5% were aware of a new law that grants people accompanied by an assistance dog broad admission rights to public spaces, including HCFs. With an expected further increase in popularity of AAIs high quality standards which include infection control measures and animal welfare should be adopted by all AAI providers and recipients. This will ensure a safe implementation of this complementary medicine, where both sides – the human and the animal – can benefit.

## 1 Introduction

In the public opinion as well as under professionals working in the field of human-animal interactions (HAIs), there is wide consent that humans benefit from interactions with animals [[1], [2], [3]]. Consequently, animal-assisted interventions (AAIs) are constantly increasing. The International Association of Human-Animal Interaction Organizations (IAHAIO) defined AAI as “a goal-oriented intervention that intentionally includes or incorporates animals in health, education and human services (e.g. social work) for the purpose of therapeutic gains in humans” [4] and subsumed the terms animal-assisted therapy (AAT), animal-assisted education (AAE) and under certain conditions animal-assisted activities (AAA) like animal visits. Research in this field has increased exponentially during the past decades. However, the scientific literature is not always clear on the efficacy of AAIs - sometimes even showing contradictory results [5,6]. Nonetheless, there is a growing body of evidence that positive effects can be scientifically proven by well-designed clinical trials [7].

Besides potential benefits, interactions with animals may also be associated with certain risks for humans. These include accidents (e.g. dogs jumping at people), injuries by scratches and bites, allergies and the transmission of zoonotic pathogens via direct or indirect contact or by vectors like fleas and ticks. On the other side, the animals' health might be at risk by transfer of pathogens, overload or inappropriate handling by AAI recipients. It is consent under HAI professionals that the positive effects of HAIs outweigh the potential harms to animals and humans. However, the above-mentioned hazards should not be neglected, especially when vulnerable individuals like infirm persons or people with an impaired immune system are involved. Several international and national AAI guidelines and recommendations [1,3,[8], [9], [10]] include measures to mitigate or eliminate these risks, but no legal regulations exist for AAI in Germany to date. Since it is unclear to what extent animal handlers follow existing recommendations, an online survey among providers of AAI and a telephone interview in hospitals were initiated to gain knowledge about the structure, characteristics and frequency of AAIs in facilities in Lower Saxony. Lower Saxony is a state in northwestern Germany. Its round eight million inhabitants represent approximately 10% of Germany's total population. Focusing on hygiene measures in HCFs, the goal was to learn more about implemented standards and to identify possible gaps that need to be addressed.

## 2 Methods

### 2.1 Online survey

Target population of the survey were providers of AAI. A web-based questionnaire was generated and distributed via the social network of the “Federal Association for Animal-Assisted Interventions” (Bundesverband Tiergestützte Intervention e.V., BTI). Additionally, we used publicly available websites to find contact details of potential candidates for the survey. Keywords were “therapy dog”, “animal assisted (occupational) therapy, psychotherapy, logopaedics” in conjunction with the main cities in the districts as well as with postal codes to restrict the search to the region of Lower Saxony. The potential participants received a personal invitation via e-mail including a link to the online survey and a description of the purpose and the confidential nature of the survey. An e-mail reminder was sent approximately three months later. Participants were asked for their occupation, their AAI-related qualification and working field (AAT, AAE, AAA, animal visits). Further questions asked for the included animal species and the kind of facility visited (hospital, rehab hospital, nursing home, preschool/school, own property e.g. practice, farmyard) as well as for information about handling of the animals (e.g. regular vet visits, feeding). Furthermore, information on the providers' policies of the animal-human contacts was inquired.

The study was conducted during the Sars-CoV-2-pandemic (December 2020 to March 2022) which largely interfered with the participants work due to extensive restrictions (e.g. admission restrictions of health care facilities, school closures). Participants were therefore asked to give answers that reflected their status prior to the pandemic.

### 2.2 Hospital survey

Target population of the second survey were all hospitals listed in the official hospital register of Lower Saxony for the year 2021. Hospitals were interviewed by phone between January and May 2022. Again, the status “prior Covid” was evaluated. Facilities were asked whether they had experiences with animal-assisted therapies or animal visits, keeping of animals, patients bringing their animals (pets or assistance dogs) into the hospital and whether they had regulations for these animal contacts in their hygiene plan or in other policies. Further details included animal species, person performing AAI (hospital staff or independent service provider), and visited hospital departments. Additionally, the awareness of a new law (German: “Teilhabestärkungsgesetz”) that came into effect in 2021 was evaluated. Among other regulations, this law manifests admission rights to public spaces (including HCFs) for people accompanied by an assistance dog. In German law (BGG, §12e) an assistance dog is a specially trained and certified dog that lives with and supports a handler with disabilities. This definition is similar to the definition of an assistance animal by Howell et al. [11].

### 2.3 Data analysis

The online survey was conducted via the platform “LamaPoll” [12]. Raw data were exported from LamaPoll to MS-Excel 2016 (Microsoft Corporation, Redmond, WA, USA). Data from the telephone survey were entered into an MS-Excel spreadsheet. For both surveys MS-Excel 2016 was used to generate descriptive data.

## 3 Results and discussion

### 3.1 Online survey's participants' working field and qualification

85 responses from 305 contacted individuals could be included in the data analysis. A total of 82% of the respondents stated that they provide AAIs in an occupational context, 13% were both occupationally and voluntary active, and only 5% worked solely on a voluntary level. These numbers show that measures taken to distribute the questionnaire mainly reached individuals providing AAIs in a professional context. Individuals that provide voluntary services without being organized in associations and maintaining internet platforms were likely not reached by our efforts. Twenty-nine percent of the respondents provide their services in HCFs (i.e. hospital, rehab hospital, nursing home), 53% work in preschools or schools and 46% named their own property (i.e. practice, farmyard) as working location. “Other location” was chosen by 24% and was in most cases specified as private households or supervised accommodations. Twenty-four percent provided their services in more than one type of location.

Due to the fact that there is no nationally recognized accreditation program in Germany and terms like “Animal-Assisted Intervention Specialist” (German: “Fachkraft für tiergestützte Interventionen”) are not protected, the AAI-related qualification of the participants was not easy to rate. As curricula accredited by the organizations ISAAT (International Society for Animal Assisted Therapy) and ESAAT (European Society for Animal Assisted Therapy) are widely recognized, these qualifications were regarded as high standard. Sixty-nine percent of all respondents (*n* = 72) had at least a therapy-oriented training as “Human-Dog-Team for therapy”. Among people working in HCFs, this was the case for almost all respondents (19/20), and 55% had the highest qualification “Animal-Assisted Intervention Specialist”. In all but two cases these were accredited by ISAAT or ESAAT. This indicates a high standard of qualification in this study collective which might reflect the above-mentioned selection of respondents.

### 3.2 Animal species

**Fig. 1:**
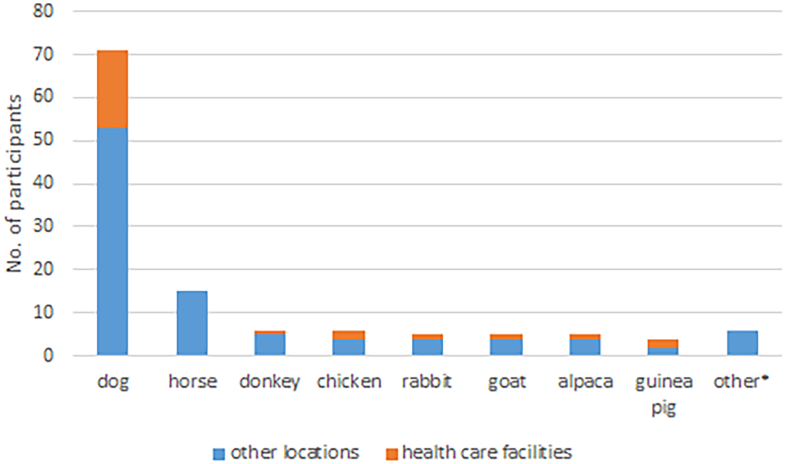
Animal species used in AAIs. *Other (*n* = 1): sheep, pig/minipig, koi carp, cat, ox, turtle.

Dogs were by far the predominant species involved in all fields of AAI (86% of providers) and for 66% this was the only species they worked with. Thirty percent exclusively worked with dogs in preschools or schools (School Support Dog) while 23% (*n* = 18) worked with dogs in HCFs. Beside dogs, this group of respondents also named donkeys, chicken, rabbits, goats, alpacas and guinea pigs (Fig. 1). Single mentions included sheep, pig/minipig, koi carp, cat, ox and turtle. All of the latter were attributed to the location “own property/practice”. This species list qualitatively and quantitively resembles the one reported by Otterstedt [13]. Similar results were found by Schuurmans et al. [14] for animals involved in AAIs in nursing homes. According to various guidelines only domesticated species that are adapted to live and socially interact with humans are suitable for AAIs [4,15]. Besides animal welfare, this requirement is based on the fact that contact to wild and exotic species might harbor a higher risk of transmission of zoonotic pathogens [16,17]. In agreement with these guidelines only a single exotic animal, a turtle, was mentioned. In addition, an AAI where this animal is only observed in its environment without close contact [15] would also be acceptable.

### 3.3 Veterinary care

The majority of respondents (61%) had arranged a schedule with their vet for regular examinations and prophylactic treatments. Among all dog owners, 63% had scheduled regular vet visits, but only 39% of those working in HCFs had done so. As shown in Table 1, almost all dog owners (96%) fulfilled guideline requirements of at least yearly health evaluations [3,9,10]. Remarkably, although 23% of dogs saw the vet at least quarterly, the dog's behavior and fitness for the special task (mentally and physical) seemed to be no common subjects of these evaluations. The majority of dog owners dewormed their dogs quarterly which is more frequent than the average deworming rate of 2.07 times per year found in the overall pet dog population in Germany [18]. Although sometimes additional faecal examinations were performed, none of the participants performed monthly deworming/testing, which the European Scientific Council Companion Animal Parasites (ESCCAP) suggests for professional dogs when the prevention of shedding of worm eggs/larvae is the target [19]. Nineteen percent of all dog owners only dewormed their dog when the faecal examination was positive. This habit is risky due to the suboptimal sensitivity of the classical faecal examination. Half of all dog owners performed prophylaxis against ectoparasites regularly during the season. One third protected their dogs all year round, which is advisable at least for some parts of Germany as in recent years ticks were also active during mild winter periods [20]. Tablets were the most common product used for tick/flea prophylaxis. The application of tablets is convenient, and most products provide long-term efficacy. However, these systemically active substances do not provide repellent efficacy and, therefore, these dogs might be at a higher risk for mechanical transfer of ticks. Of special concern is that several owners reported the use of substances without scientifically proven efficacy (e.g. feed additives, coconut oil, homeopathy) against ectoparasites. In three cases these were the only treatments the dogs received – two of these dogs worked in HCFs.

**Table 1:** Details of veterinary examinations and treatments.

	No. (%) of respondents with tasks in HCFs [*n* = 17]	No. (%) of respondents with tasks in other locations [*n* = 52]
Frequency of vet checks		
≥ quarterly	4 (24)	12 (23)
2×/year	8 (47)	23 (44)
yearly	4 (24)	15 (29)
Every 2 years	0 (0)	0 (0)
Irregular	0 (0)	2 (4)
Other	1 (6)	0 (0)
Items of vet checks		
General health examinations	16 (94)	51 (98)
Behavior test	2 (12)	5 (10)
Fitness for the task (with relation to health and age)	7 (41)	27 (52)
Faecal examination for endoparasites	8 (47)	21 (40)
Vaccination (when needed)	17 (100)	51 (98)
Frequency of deworming		
Only when faecal exam is positive	2 (12)	11 (21)
Yearly	0 (0)	3 (6)
2×/year	3 (18)	3 (6)
Quarterly	12 (71)	35 (67)
Monthly	0 (0)	0 (0)
Treatment/prophylaxis against ectoparasites (fleas and ticks) -scheme		
Only when dog is infested	4 (24)	5 (10)
Regularly during the season	6 (35)	29 (56)
All year round	7 (41)	16 (31)
Other scheme⁎	0 (0)	2 (4)
Treatment/prophylaxis against ectoparasites (fleas and ticks) -products		
Spot-ons	5 (29)	21 (40)
Collars	5 (29)	7 (13)
Tablets	8 (47)	34 (65)
Shampoo	4 (24)	4 (8)
Powder	0 (0)	0 (0)
Other⁎⁎	4 (24)	6 (12)

⁎ Other schemes: longer intervals between treatments in winter.

⁎⁎ Other products included feed additives like brewer's yeast, black cumin oil, commercial plant based feed additives and homeopathy as well as coconut oil for external use.

### 3.4 Dog maintenance

The majority of dogs (59%) was acquired from a breeder. Twenty-one percent were taken over from private dog owners and 8% stemmed from the respondent's own breeding. Ten dogs (12%) were received from animal welfare organizations. Seventy percent of dogs were purchased at an age of up to 16 weeks and 17% were older than one year at the time of acquisition. The most effective time point for socialization and habituation is the age up to 16 weeks. In adult dogs it might be more difficult or might take longer to get them used to new situations. This could be especially relevant for rescue or shelter dogs, whose early socialization and habituation might be unknown. In addition, dogs from abroad (*n* = 7 in this study) might harbor zoonotic pathogens that are not autochthonous in Germany.

Most AAI guidelines prohibit the feeding of raw meat to dogs involved in AAIs. In the presented study 22% of all dog owners fed BARF (Biologically Appropriate Raw Food) diets to their dogs. The vast majority of dogs (82%) received at least one critical feed category (e.g. raw meat, dehydrated but otherwise raw feed, meaty treats). The popularity of feeding raw meat and meaty treats is not limited to Germany. Discrepancies between guideline recommendations and the policies of therapy animal organizations have been described in the United States [21,22]. These surveys showed that only 13 to 25% of therapy animal organizations prohibit raw meat diets. Given that these diets are regularly contaminated with zoonotic pathogens like *Salmonella* spp. [23] or bacteria harboring genes for antibiotic resistance [24], and that there is no scientific proof that raw diets are healthier, it is unclear why so many dog owners do not follow the guidelines.

### 3.5 Hygiene measures

Seventy-one percent of all participants confirmed to have a written policy they adhere to. Eighty-two percent of these policies contained specifications regarding the clients' correct behavior, and 57% specified inclusion/exclusion criteria for clients (e.g. allergies). Ninety percent included details for the animal's health monitoring and 80% defined animal welfare standards. Hygiene requirements for the animal were defined by 92%, and 63% set standards for the documentation of their visits or therapy sessions.

**Fig. 2:**
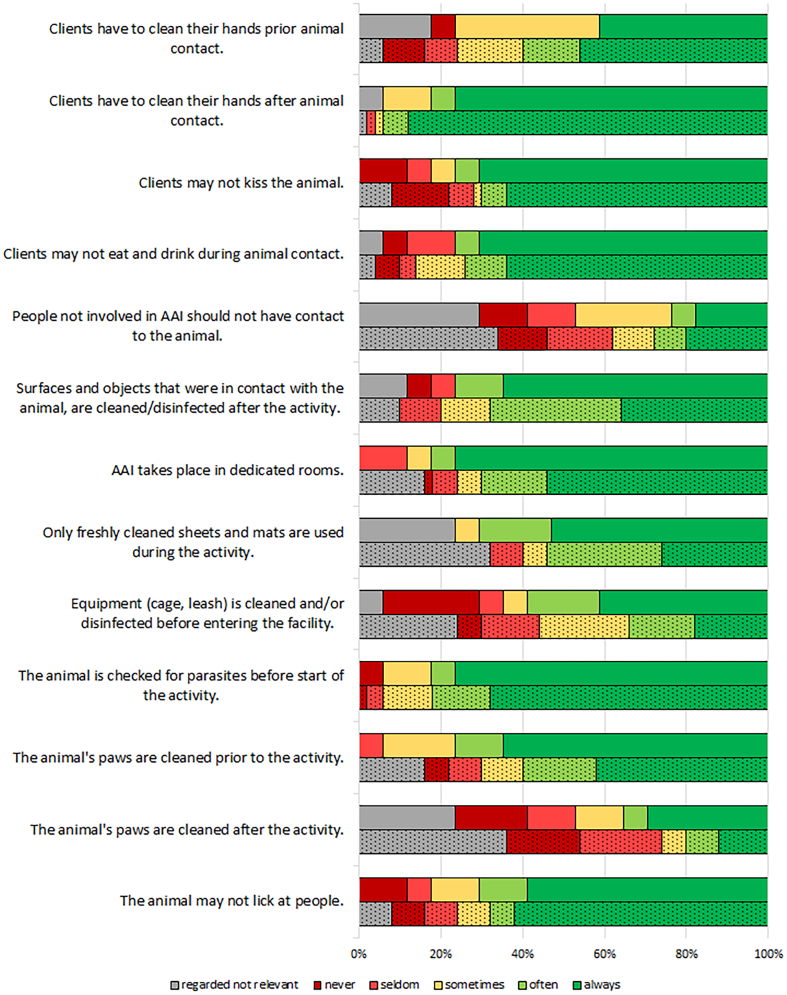
Hygiene measures. Upper bar (full color): respondents with tasks in HCFs; Lower bar (dotted): respondents with tasks in other locations.

Adherence to hygiene measures by dog owners is displayed in Fig. 2. The discrepancy before and after animal contact is remarkable. While the client's hand hygiene was obligatory after animal contact for the majority of respondents (85% with statement “always”), only 45% let their clients always clean their hands before animal contact. The same applied regarding the dog's paws. Cleaning the paws prior to the intervention is much more common than afterwards. This leads to the assumption that the dog handlers are not aware of a possible pathogen transfer from clients or from the environment to the dog, or that they estimate the risk to be low. In a significant number of cases, the necessity of using clean equipment (e.g. the dog's leash, mats and sheets) during AAI was regarded as not relevant, or equipment was not regularly cleaned. In addition, other measures that are considered best practice by the majority of guidelines, like prevention of kissing the animal, prohibiting eating and drinking during animal contacts and preventing the dog from licking the client, were also not always regarded as mandatory. These data show that professional therapy dog handlers are not always aware of the special situation in HCFs.

The majority (95%) of respondents performed AAI tasks three or less times per week in up to five different facilities per month (Table 2). In all cases, the animal had contact to more than one client per day. These data show that a risk of pathogen transmission might not only been given between clients in the same facility but also between different facilities. This emphasizes the importance of proper hygiene measures after every contact to clients. Specializations of visited hospitals were psychiatry and psychosomatic medicine and “others” without providing details.

**Table 2:** Details of AAIs in health care facilities.

	No. of respondents (%)
Frequency	
All workdays	1 (5)
2–3×/week	9 (47)
1×/week	5 (26)
1×/month	3 (16)
<1×/month	1 (5)
Number of different facilities visited with the same animal per month	
1	8 (42)
2-5	10 (53)
6-10	1 (5)
>10	0 (0)
Length of animal contact per client (hours)⁎	
≤0.5	6 (33%)
≤1	11 (61%)
≤2	1 (6%)
>2	0 (0%)
Number of different clients per animal per day⁎	
1	0 (0%)
2-3	9 (50%)
4-10	8 (44%)
>10	1 (6%)

⁎ One response missing.

**Fig. 3:**
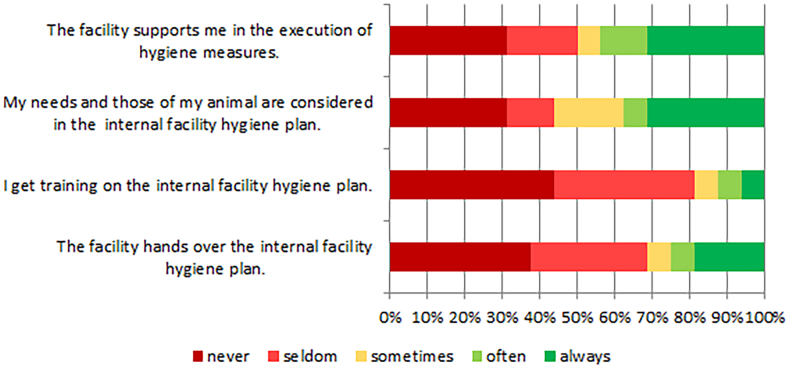
Dealing with the internal facility hygiene plan.

Limiting the length of animal contact is necessary to prevent the animal from being overworked. All but one respondent limited the length to one hour or less, which is in accordance with most guidelines [9,10]. The cooperation between animal handlers and facilities in terms of hygiene measures needs attention. The majority of animal handlers felt not supported by the facilities. Neither was the animal handler trained on the internal facility hygiene plan, nor was the hygiene plan handed over (Fig. 3).

### 3.6 Hospital survey

Of 166 hospitals in Lower Saxony 148 (89%) participated in the study. In 88% the hospitals' infection control personnel answered the questions. Eighty-one (55%) of the participants reported that at least one type of animal contact (AAT, animal visit, assistant dogs or patients' pets) was allowed in their facility, but in only 28% AAT and animal visits took place on a regularly basis (Table 3). Dogs were again the predominant species involved in AATs and visits. Four hospitals mentioned visits of further species (alpacas, rabbits, guinea pigs, chicken and snails). Animal species kept by the facilities included fish (aquarium), cats, rabbits and sheep. In one case, the facility kept wild boars, ponies, alpacas, donkeys, geese, chicken and ducks. In the majority of cases (*n* = 30, 73%) members of the hospital staff conducted the AATs and visits. These numbers show that AAIs are more common in hospitals than expected from the results of the online survey, probably due to the fact, that the survey did not cover this group of animal handlers.

**Table 3:** Results of the hospital survey.

Type of animal contact	No. (%) of hospitals	Standards present No. (%) of hospitals
Hospital has experiences with AAT / animal visits	41 (28)	32 (78)
Hospital keeps animals	9 (6)	4 (44)
Hospital allows assistance dogs / pet animals	52 (35)	41 (79)

Hospital wards and departments mentioned in the context of AAT and animal visits comprised psychiatry, psychosomatic medicine, pediatrics, geriatrics, dementia units and palliative care. Overall, AAI seems to be less frequently performed in German hospitals compared to the US [9] or Canada [25], especially regarding acute care settings like internal medicine wards. Fifty-two (35%) hospitals allowed assistance dogs or visits of patients' pets. Pet visits were almost exclusively single events (e.g. “last visits” in palliative care units). Three psychiatric hospitals allowed their patients to bring their pets (dogs, small rodents). Approximately one fifth of hospitals providing AAIs/animal visits lacked standards for the execution of this complementary therapy. Only eight hospitals (5%) were aware of the new legislation regarding admission rights for people accompanied by assistance dogs, showing a large need to implement these regulatory requirements.

## 4 Conclusion

Our studies reveal that AAIs take place in Lower Saxonian health care facilities, although to a much lesser extent than what is common in other countries. One limitation of this study is that it was conducted during the Sars-CoV-2-pandemic and answers referred to the pre-pandemic situation. The retrospective information provided might have been less accurate than a survey on current practice. In hospitals, mainly members of the staff conduct AAIs, bringing their pets to the HCF. Nevertheless, a relevant number of occupational AAI providers stated to perform at least part of their work in HCFs. It was shown that many of these AAI providers deviated from what various guidelines have defined to be best practice. These include important basic hygiene procedures and other infection control measure like the prohibition of raw meat diets. Overall, the handling of therapy dogs often reflected that of a “normal” pet ownership, not considering the special needs of HCFs which often host vulnerable individuals. Due to their expertise on zoonoses, a broader involvement of veterinarians in the field of AAI is essential. The role of veterinarians is, however, not limited to infectious diseases: Regular health monitoring of animals involved in AAI should be performed to ensure that the animals are physically able to cope with the specific demands of AAI. Ideally, the animals' well-being should also be evaluated regularly during AAI to ensure that animals are not overburdened. Thus, the role of veterinarians in AAI is not only to maintain the animal's health, but also to educate the stakeholders. In most cases, HCFs do not provide appropriate guidance to the AAI providers. Thus, with an expected further increase in popularity of AAI, improved communication with AAI providers as well as the establishment of high-quality standards including infection control measures and animal welfare should be adopted. This will ensure a safe implementation of this complementary medicine, where both sides – the human and the animal – can benefit.

In addition, the new admission right for people accompanied by an assistance dog needs to be implemented. Although similar regulations have been in place in other countries for decades, this demand might imply a paradigm shift in some German institutions.

## Funding

This project (no. 01KI1814) has been funded by the 10.13039/501100002347Federal ministry of Education and Research within the network of the German Research Platform for Zoonoses.

## Declaration of Competing Interest

None.

## Data Availability

Data will be made available on request.
